# The Statistics of *q*-Statistics

**DOI:** 10.3390/e26070554

**Published:** 2024-06-28

**Authors:** Deniz Eroglu, Bruce M. Boghosian, Ernesto P. Borges, Ugur Tirnakli

**Affiliations:** 1 Faculty of Engineering and Natural Sciences, Kadir Has University, Istanbul 34083, Turkey; deniz.eroglu@khas.edu.tr; 2 American University of Armenia, Yerevan 0019, Armenia; bruce.boghosian@tufts.edu; 3 Department of Mathematics, Tufts University, Medford, MA 02155, USA; 4 Instituto de Fisica, Universidade Federal da Bahia, Rua Barão de Jeremoabo, Salvador 40170-115, Brazil; ernesto@ufba.br; 5 National Institute of Science and Technology of Complex Systems, Rua Xavier Sigaud 150, Rio de Janeiro 22290-180, Brazil; 6 Department of Physics, Faculty of Arts and Sciences, Izmir University of Economics, Izmir 35330, Turkey

**Keywords:** *q*-Statistics, nonextensive statistical mechanics, generalized entropies, complex networks

## Abstract

Almost two decades ago, Ernesto P. Borges and Bruce M. Boghosian embarked on the intricate task of composing a manuscript to honor the profound contributions of Constantino Tsallis to the realm of statistical physics, coupled with a concise exploration of *q*-Statistics. Fast-forward to Constantino Tsallis’ illustrious 80th birthday celebration in 2023, where Deniz Eroglu and Ugur Tirnakli delved into Constantino’s collaborative network, injecting renewed vitality into the project. With hearts brimming with appreciation for Tsallis’ enduring inspiration, Eroglu, Boghosian, Borges, and Tirnakli proudly present this meticulously crafted manuscript as a token of their gratitude.

## 1. Introduction

Statistical physics has profoundly enhanced our understanding of nature, life, and physical phenomena by revolutionizing our comprehension of macroscopic phenomena—the observable properties of materials—through their connection to microscopic laws, which govern the behavior of individual atoms and molecules. This bridging of the microscopic and macroscopic worlds has enabled scientists to predict and explain macroscopic phenomena such as temperature, pressure, and entropy based on the statistical behavior of microscopic constituents [1,2]. Statistical physics has provided a robust microscopic foundation for thermodynamics. The statistical interpretation of entropy, in particular, has deepened our understanding of the second law of thermodynamics and the direction of natural processes [3]. The field has greatly advanced our knowledge of phase transitions, such as the change from solid to liquid or liquid to gas, explaining critical phenomena and the nature of phase changes through concepts like critical exponents and scaling laws [4]. In the quantum realm, these principles have been extended to systems governed by quantum mechanics, elucidating phenomena such as superconductivity, superfluidity, and the behavior of Bose–Einstein condensates [5]. Concepts like entropy have also become fundamental to understanding information processing and transmission, leading to applications in information theory and computational complexity [6]. In materials science, statistical physics has provided insights into the properties of new materials, such as polymers, glasses, and complex fluids, driving the development of new technologies and materials with tailored properties [7,8]. On the largest scales, statistical physics has contributed to our understanding of the structure of the universe, the distribution of galaxies, and the thermodynamic history of the cosmos, including the study of black holes and the early universe [9]. Moreover, the methods and concepts of statistical physics have influenced various other disciplines, including chemistry, biology, and economics. In biology, for example, it aids in understanding processes like protein folding and the behavior of biological membranes [10]. Overall, statistical physics has transformed physics by providing a powerful framework to understand and predict the behavior of complex systems, making it an essential pillar of modern science [11].

Statistical physics is indeed largely founded upon the framework of Boltzmann–Gibbs (BG) statistics, which emerged in the late 19th century. Ludwig Boltzmann’s introduction of entropy and its correlation with the microscopic states of a system laid the groundwork for statistical mechanics [12]. Subsequently, Josiah Willard Gibbs expanded upon Boltzmann’s ideas, formulating a more rigorous mathematical framework known as Gibbs statistical mechanics [13]. This framework became instrumental in relating the macroscopic properties of a system, such as temperature, pressure, and energy, to the microscopic configurations of its constituent particles. Boltzmann–Gibbs statistics provides a robust framework for describing the behavior of large systems composed of numerous particles. Considering the statistical distribution of these microscopic states enables the calculation of thermodynamic quantities and the prediction of macroscopic behavior. However, Boltzmann–Gibbs statistics alone was insufficient to address the entire puzzle of bridging the macroscopic and microscopic worlds. In certain non-equilibrium and complex systems contexts, Boltzmann–Gibbs statistics exhibit limitations. To address these challenges, Constantino Tsallis proposed a generalization of the BG formalism, known now as *q*-Statistics, which is based upon a non-additive entropy having the BG entropy as a special case. These kinds of generalized entropies have already been introduced in the literature, particularly within the context of information theory [14], but they had never been used for the generalization of statistical mechanics. Tsallis’ *q*-Statistics offer a broader framework applicable to systems with long-range interactions and non-equilibrium dynamics, providing insights into phenomena beyond the scope of traditional statistical mechanics [15,16].

The well-deserved success of statistical physics is not only evident in its transformative insights into the behavior of complex systems, but also in the robust acknowledgment it receives through scholarly citations. In the scientific community, acknowledgment of scholarly contributions is quintessentially expressed through citations, encapsulating the cumulative impact of scientific endeavors. The statistics of these citations serve as barometers of influence within the scientific world, reflecting the reach, significance, and enduring legacy of research contributions. This quantitative analysis of scientific citations and related data falls within the purview of scientometrics, which aims to objectively quantify the impact of scientific outcomes, whether in the form of scientific papers or the corpora of an individual scientist or even a group of scientists. Despite the inevitable inaccuracies and biases inherent in these measures and methods, various indexes have gained widespread usage among the scientific community and funding agencies. It is implicitly assumed that such indexes can capture universal behaviors, at least within specific domains, such as scientific fields.

In this article, the collective measures of *q*-Statistics, a branch of statistical physics initiated by Tsallis’ landmark 1988 paper [15], are examined. Additionally, Tsallis’ collaboration network, which significantly shaped the *q*-Statistics community, is reconstructed to understand how *q*-Statistics has spread globally and been influenced by key contributors. Functions emerging within this area provide statistical descriptions of the field’s time evolution and the geographical distribution of contributors, among other metrics. The outcome of the analysis shows the remarkable success of the field and its strong impact on the scientific community. While these observations are notable, they align with the broader understanding that numerous complex phenomena in the physical, biological, computational, and social domains exhibit behaviors accurately described by *q*-Statistics.

## 2. Diffusion of *q*-Statistics Ideas

Aiming to understand natural phenomena through statistical approaches has extended into the intriguing research field of statistically analyzing complex social systems by identifying the distributions within a given context. First, attention is specifically drawn to two particular examples of the diffusion of ideas within a social community, focusing on their “success”:

(i) *The distribution of the number of citations of scientific papers.* In ref. [17], this scientometric feature was addressed, and it was concluded that highly cited papers follow a power-law distribution, while low-cited papers follow a stretched exponential distribution, suggesting that different phenomena govern these two regimes. In ref. [18], it was found that the same data could be represented by a single distribution, namely, a q-exponential distribution:
  (1) $$\exp_{q}\left(x\right)\equiv {\left[1+(1-q)x\right]}_{+}^{1/(1-q)}\;\;\;\;\;\;\left(q\in R\right)$$
  where ${\left[\cdots \right]}_{+}$ means that $\exp_{q}\left(x\right)=0$ if [1+(1−q)x]≤0. This finding suggests that both high- and low-cited papers may follow the same rules;
(ii) *The distribution of the number of weeks that pop musicians stay in Britain’s top-selling lists.* In ref. [19], the top-75 best-selling musicians on a week-by-week basis from 1950 to 2000 in the UK were analyzed, and it was found that a stretched exponential can fit the data. In ref. [20], it was shown that the same data could be equally well-fitted with a function that displays an intermediate power-law regime and presents a crossover to an exponential tail. This function, introduced by [21] within a different context (reassociation of carbon monoxide in folded myoglobin), is given by
  (2) $$f\left(x\right)\equiv {\left[1-\frac{\beta_{q}}{\beta_{1}}+\frac{\beta_{q}}{\beta_{1}}e^{\left(q-1\right)\beta_{1}x}\right]}^{1/(1-q)}\;\;\;\;\;\left(\beta_{q}>\beta_{1}\geq 0;\;q>1\right)\;,$$
  that is, a generalization of the *q*-exponential, as it reduces to $f\left(x\right)=\exp_{q}\left(-\beta_{q}x\right)$ in the limit $\beta_{1}\to 0$.

These two examples of a measure of success that can be represented by *q*-exponentials or functions that belong to the family of *q*-exponentials support a conjecture that these social phenomena have a nonextensive nature.

In the present work, the growth and spread of nonextensive ideas among scientists are considered as an instance of the diffusion of knowledge within a social community. The time evolution of the number of papers on *q*-Statistics (including printed or electronic papers, books, theses, etc.) is regarded as the dynamical aspect of the diffusion process. The scientific community is viewed as a “phase-space” of the system. The geographical distribution of the scientists represents a measure of the filling of the phase-space. The spatial spread is indicated by the distribution of countries to which the authors of those papers belong. Country rank one is assigned to the country with the highest number of different authors within the *q*-Statistics literature. Different statistical measures of the diffusion of *q*-Statistics are found, with some satisfactorily described by *q*-exponentials or functions belonging to the *q*-exponential family.

**Figure 1:**
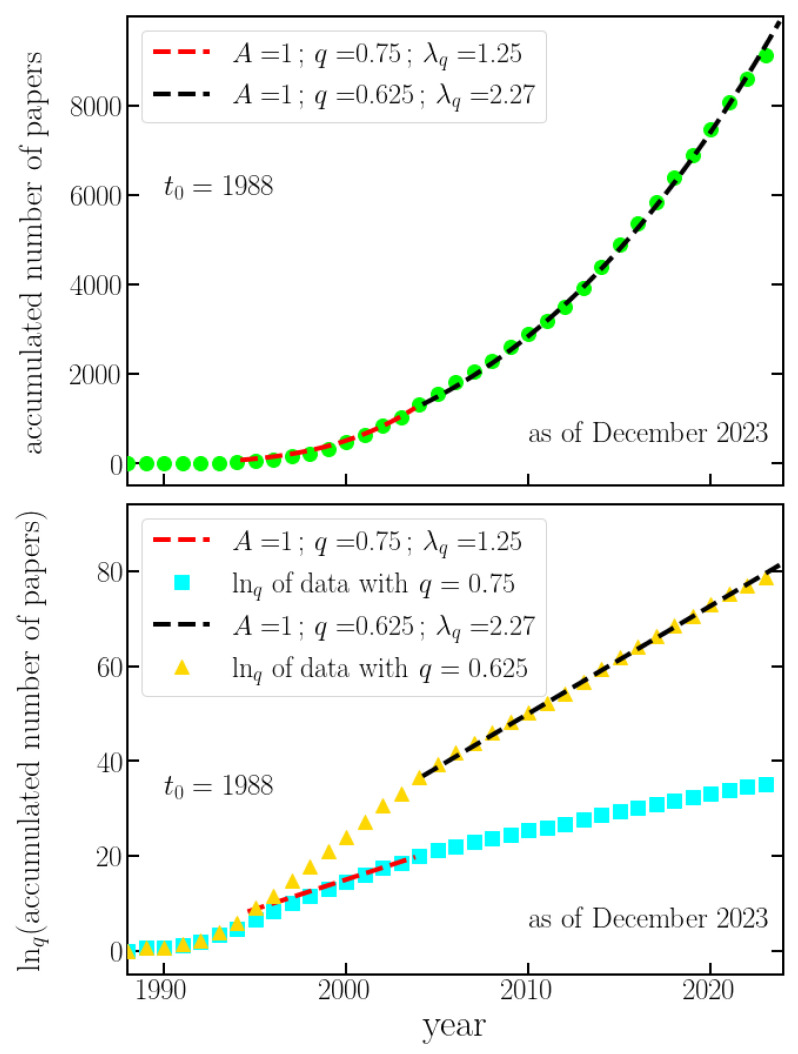
**Cumulative number of published papers per year.** The cumulative number of papers related to *q*-Statistics published each year (as of December 2023) is depicted, where the data follows two distinct *q*-exponential regimes ($f\left(t\right)=A\exp_{q}\left(\lambda_{q}t\right)$) for two different time spans. (**Top Panel**) The red dashed line represents the regime from 1992 to 2004 (q=0.625), and the black dashed line represents the regime from 2004 to the present (q=0.75). Each regime’s trend is well-approximated by a *q*-exponential with the parameters provided in the figure. (**Bottom Panel**) The top panel is in linear–linear scale, while the bottom panel is in mono-*q*-log scale: the ordinate is represented in *q*-log scale, with the *q*-values 0.625 (yellow triangles) and 0.75 (blue squares). The same *q*-log functions are applied to the fitting curves in the top panel, represented again by dashed red and black lines in the bottom one.

Figure 1 illustrates the cumulative number of papers per year, revealing three distinct regimes. Initially, the linear regime spans from 1988, coinciding with Tsallis’ first paper on the subject, to approximately 1992. The onset of the first *q*-exponential regime ($f\left(t\right)=A\exp_{q}\left(\lambda_{q}t\right)$, q<1, $\lambda_{q}>0$) is observed around 1992 (q=0.75), indicated by the red dashed line in Figure 1. Notably, this period marked the establishment of the connection between nonextensive statistical mechanics and thermodynamics [22], as well as the first connection to a physical system, namely, self-gravitating stellar polytropes [23]. A bibliography on the theme of *q*-Statistics has been continously updated by Constantino Tsallis since 1995. The name of the file available at the URL [24], TEMUCO.pdf, was chosen in honor of the city that held the *IX Taller Sur de Física del Sólido*, 26–29 April 1995, Misión Borea, Temuco, Chile. The two above important papers and the continuously updated bibliography likely facilitated the transition from linear growth to the *q*-exponential regime. Subsequently, a second *q*-exponential regime emerged around 2004 and persisted until December 2023, with q=0.625. It is worth noting that, to maintain analogy with the current nonextensive nomenclature, the index associated with the time evolution, $q_{sen}$, denotes sensitivity to initial conditions ($q_{sen}<1$), as depicted in Figure 1.

**Figure 2:**
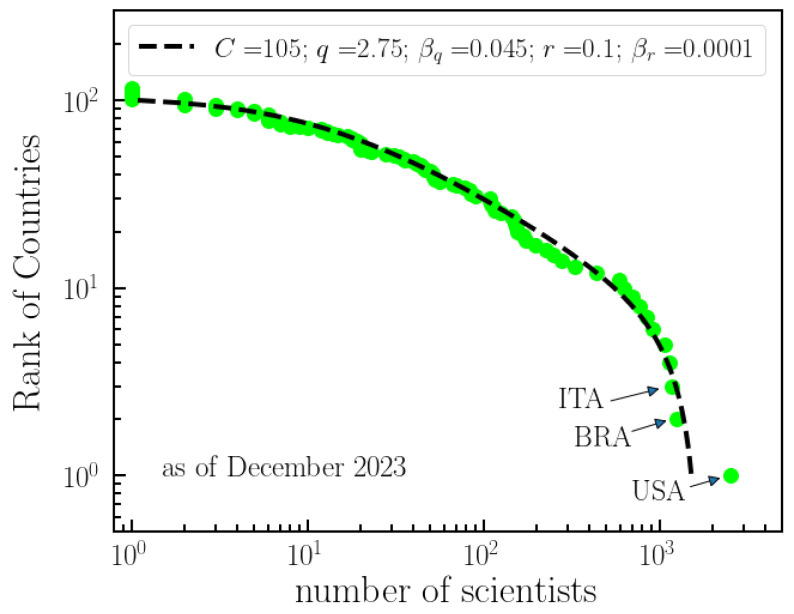
**National researcher contributions to *q*-Statistics.** The collective involvement and impact of researchers from different countries in the field of *q*-Statistics, as reflected by the number of scientists with published papers in the field. The figure illustrates the ranking of countries by the total number of scientists with published papers on *q*-Statistics. The dashed line corresponds to the fitting of the data with a (*q*,*r*)-exponential (see text), with the parameters indicated in the figure. The figure highlights the varying levels of participation and influence of researchers from different nations in advancing the understanding and development of *q*-Statistics. As of December 2023, the USA, Brazil, and Italy are the three major contributors to the field.

Figure 2 presents the (unnormalized) decreasing cumulative distribution of the number of scientists that collaborated to *q*-Statistics per country. The dashed line indicates a fitting with a generalization of a *q*-exponential with two power-law regimes. In light of [16,21], one can write

(3) $$\frac{dy}{dx}\;=\;-\beta_{r}\;y^{r}-\left(\beta_{q}-\beta_{r}\right)\;y^{q}\;\;\;\;\;\left(r\leq q\right)$$

with y(0)=1, whence

(4) $$x\;=\;\int_{y}^{1}\frac{du}{\beta_{r}\;u^{r}+\left(\beta_{q}-\beta_{r}\right)\;u^{q}}$$

can be obtained. Here, *x* denotes the number of scientists and y≡R/C, where *R* is the rank of countries. In the r=1 case, Equation (4) recovers Equation (2). Notably, a *q*-value larger than 1 is found, with q=2.75, where the index associated with geographical distributions is analogous to the index $q_{stat}$ ($q_{stat}>1$), indicating a stationary state. For a given complex system, there typically exist several indices *q*, depending on the class of properties that are being analyzed. This is frequently referred in the literature as the *q*-triplet and analogous structures [25]. One of these indices is the so-called $q_{sen}$, which is typically $q_{sen}\leq 1$. Another one of these indices is $q_{stat}$, which can be, depending on the system, either $q_{stat}\geq 1$ or $q_{stat}<1$. Further details can be found in [16].

**Figure 3:**
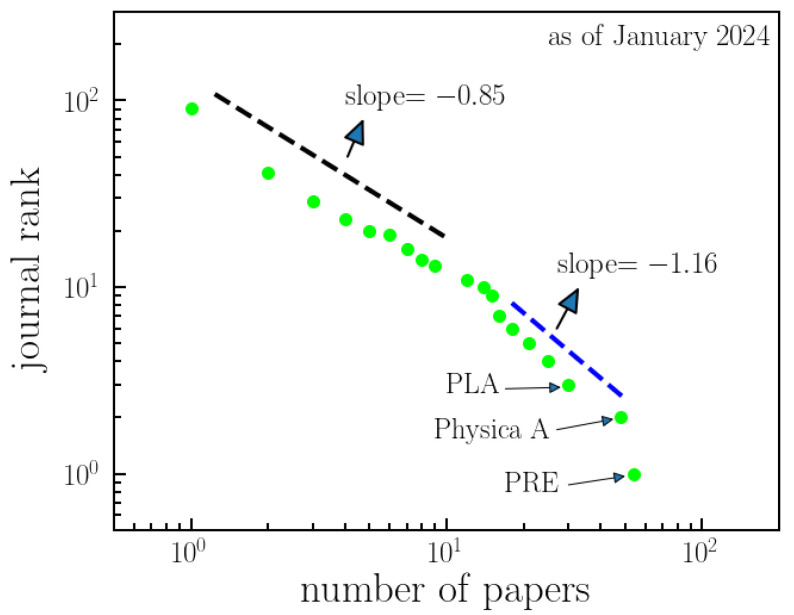
**Ranking of journals with publications in *q*-Statistics.** This figure presents the arrangement of journals based on the number of articles that they have published related to *q*-Statistics. The data exhibit two distinct power-law regimes: one for journals with a relatively small number of papers (slope = −0.85) and another for journals with a higher number of papers on *q*-Statistics (slope = −1.16). This offers an overview of the distribution of publications across different journals in the field. As of January 2024, among 91 journals, Physical Review E, Physica A, and Physics Letters A have the highest number of published papers on *q*-Statistics.

The distribution of scientific journals that have been used as vehicles for work on *q*-Statistics is shown in Figure 3. Two power-law regimes are identified, with a cross-over at about 10 papers per journal.

The statistics of *q*-Statistics indicate a *q*-exponential growth in the number of publications over the years, characterized by shifting regimes. Leading contributors to *q*-Statistics include the United States, Brazil, and Italy, with manuscripts predominantly published in impactful and longstanding journals such as Physical Review E, Physica A, and Physics Letters A. The primary architects of this success are undoubtedly Constantino Tsallis and his collaborators. Therefore, the subsequent section is dedicated to exploring the collaboration network of Tsallis.

## 3. Collaboration Network of C. Tsallis

**Figure 4:**
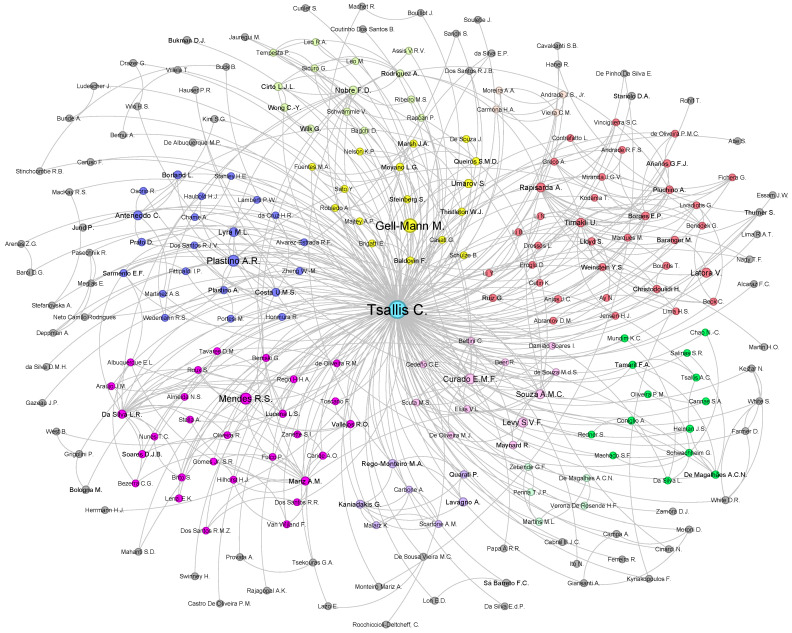
**Constantino Tsallis’ collaboration network.** Illustration of Constantino Tsallis’ collaboration network, encompassing all researchers (included in the Scopus database) who collaborated with him throughout his research career. The network comprises 236 researchers with 436 publications and 543 edges linking authors who have joint papers within the network. Node sizes are proportional to the number of citations of coauthored papers, reflecting the impact of researchers on the scientific community through their collaboration with C. Tsallis. Notably, the network reveals the presence of 11 distinct communities, each denoted by a unique color.

The collaboration network of Constantino Tsallis comprises autonomous individuals collaborating to address research problems, particularly in statistical mechanics. Remarkably, researchers are located in diverse geographic regions and represent various disciplines, including fundamental sciences, computer sciences, psychology, and even art. This dynamic collaboration network has evolved over many years, culminating in the configuration depicted in Figure 4, facilitating the sharing and dissemination of scientific knowledge. The network’s formation is not solely attributed to technological advancements but also to progress in international research and camaraderie.

Data containing joint papers, author names, and Scopus IDs were initially collected from the Scopus database (on 17 January 2024) to reconstruct Constantino Tsallis’s collaboration network. Utilizing unique Scopus IDs allowed for the differentiation of homonym author names. Notably, some significant contributions by Tsallis, such as the book titled “Nonextensive Entropy: Interdisciplinary Applications” by M. Gell-Mann and C. Tsallis, published by Oxford University Press, were absent from the Scopus database. These important contributions were integrated into the parsed Scopus data, completing the data preprocessing approach.

**Figure 5:**
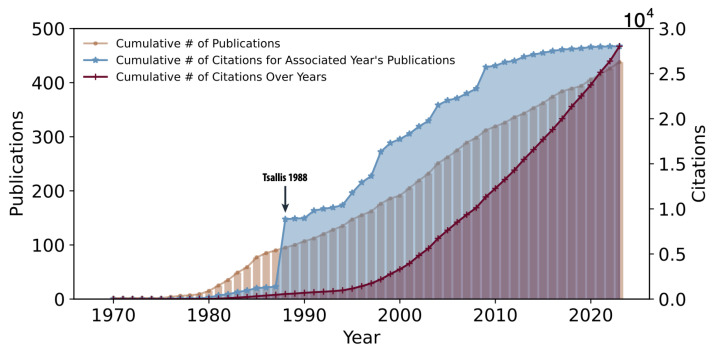
**Constantino Tsallis’ Publications and Citations.** Illustrations depict the cumulative number of publications (orange) and citations (red) throughout the academic career of C. Tsallis, spanning from 1970 to the present. Cumulative plots of the total number of citations for papers published in each respective year are also provided (blue). The seminal article by C. Tsallis on *q*-Statistics, published in 1988, stands out as a highly cited paper, marked by the arrow denoted “Tsallis 1988”.

The finalized publication record of C. Tsallis indicates a total of 438 publications and 28,039 citations. The cumulative increase in publications (orange) and citations (red) is depicted in Figure 5. Additionally, Figure 5 presents the cumulative citation distribution of papers relative to the year of publications (blue line). The most-cited paper by C. Tsallis, titled “Possible generalization of Boltzmann-Gibbs statistics” [15], is prominently highlighted by a significant increase in the blue curve, denoted by an arrow and the text “Tsallis 1988”.

Authors of coauthored articles or books were considered connected; in essence, if two authors published a joint paper, they were linked in the network structure. As the dataset encompasses all of C. Tsallis’ works, Tsallis is considered a (co)author of all the papers, thereby linked to all other researchers within the network. Links between other researchers indicate that the connected authors have joint paper(s) with C. Tsallis. Consequently, the collaboration network comprises 236 nodes, indicating that C. Tsallis has 235 coauthors and 543 links. In Figure 4, node sizes are proportional to the total number of citations received from coauthored papers with C. Tsallis. Thus, large nodes do not signify that the author has numerous papers with Tsallis; rather, they have a substantial number of citations together.

In addition, a community detection algorithm was employed to discern 11 distinct clusters, each delineated by different colors, as depicted in Figure 4. These clusters are based on the coauthorships among C. Tsallis’ collaborators, where nodes represent authors and edges represent shared publications with Tsallis. Each cluster also features leading authors; for instance, M. Gell-Mann is a prominent node in the yellow cluster, indicating that researchers in the yellow cluster share a common coauthoring basis. This applies to all other clusters, including important and impactful leading collaborators such as E.M.F. Curado, R.S. Mendes, A.R. Plastino, A. Rapisarda, U. Tirnakli, E.P. Borges, and others.

The clusters not only reflect the frequency of coauthorship, but also sometimes align with specific research fields and geographic locations. For example, distinct communities may emerge from authors working in similar research areas but are not strictly grouped by this criterion. Geographic factors also influence clustering, with authors from the same research field but different countries or continents often appearing in separate clusters. This global reach highlights the diverse and extensive nature of Tsallis’ collaborative network.

The nodes in the gray cluster contain researchers who had a few joint works with C. Tsallis, and they have no significant collaborations with the rest of the network to be assigned to a specific community. Although intuitively detectable from the network visualization, these insights underscore the broad and varied impact of Tsallis’ collaborations across different research fields and international borders. This information has now been incorporated to provide a clearer understanding of the community structures within the network.

**Figure 6:**
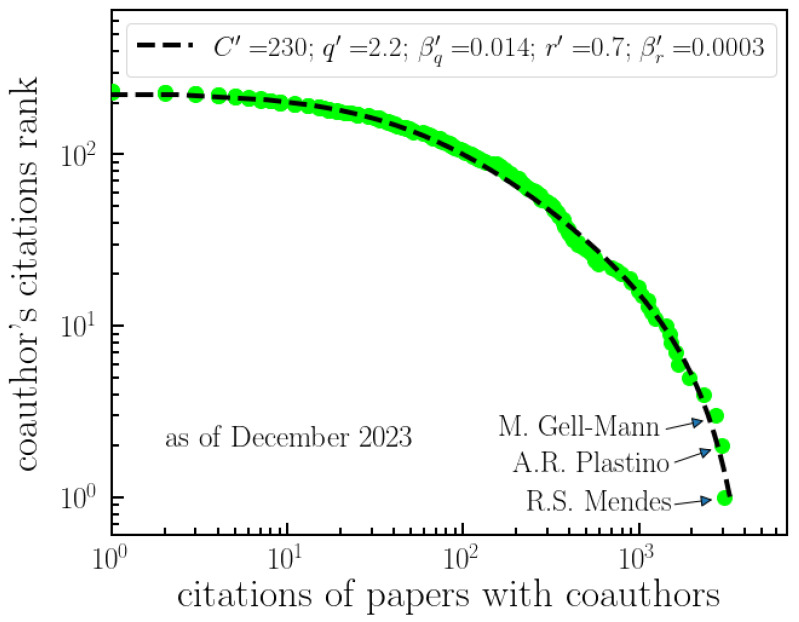
**Ranking of Tsallis’ citations with coauthors.** The analysis of citations received by articles authored by Tsallis in collaboration with other researchers. This figure displays the unnormalized decreasing cumulative distribution of citations for papers with coauthors. The data are fitted with a (*q*,*r*)-exponential model, with parameters indicated by Equations (3) and (4). The figure involves ranking these citations based on the number of times they have been cited, providing insights into the impact and influence of Tsallis’ collaborative work. The top-three most-cited coauthors with joint papers, Mendes, Plastino and Gell-Mann, are indicated.

Figure 6 shows the distribution of citations with coauthors—equivalent to the node sizes in Figure 4. In order to fit the data, we once again utilize Equations (3) and (4), where *x* now represents the citations of papers and $y\equiv R/C^{\prime }$, with *R* denoting the coauthor’s citations rank. As the number of citations is significantly larger than the number of papers, authors, or countries, the distribution is more saturated. Consequently, we were able to fit it with a single distribution with better accuracy.

**Figure 7:**
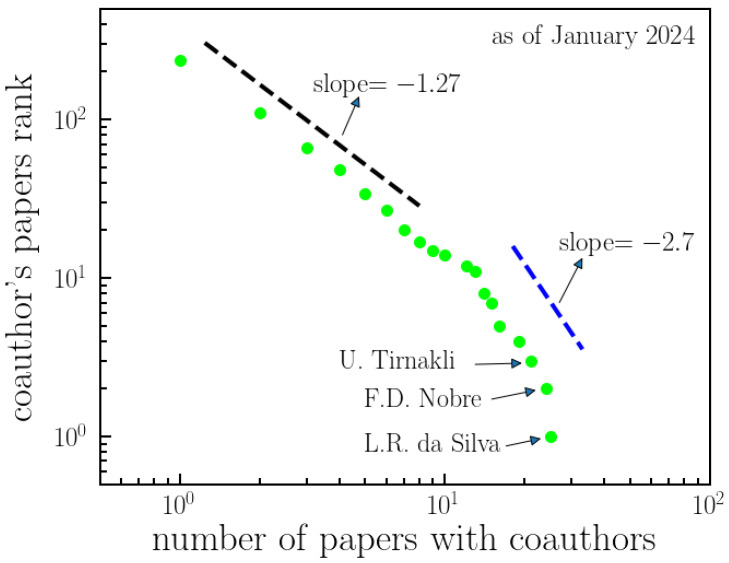
**Ranking of Tsallis’ papers with coauthors.** This analysis focuses on articles authored by Tsallis in collaboration with other researchers, specifically examining the frequency of coauthorship. The figure illustrates the unnormalized decreasing cumulative distribution of the number of papers authored by Tsallis in collaboration with others. It identifies two distinct power-law regimes, with a transition regime between them (slopes indicated). This ranking offers insights into the collaborative research efforts involving Tsallis. Additionally, the figure highlights the top-three most-frequent collaborators: da Silva, Nobre, and Tirnakli.

The distribution of the number of papers with coauthors is illustrated in Figure 7. Unlike Figure 6, this distribution is not described by a *q*-exponential function. Instead, it exhibits two distinct power-law regimes with a transition point between them. The number of joint papers with a given coauthor is influenced by different factors than the number of citations of those joint papers. Psychological or personal aspects, such as friendship or proximity, among others, may play a more significant role in shaping the behavior observed in Figure 7 compared to Figure 6, as citations are generally less personal than collaborations.

**Figure 8:**
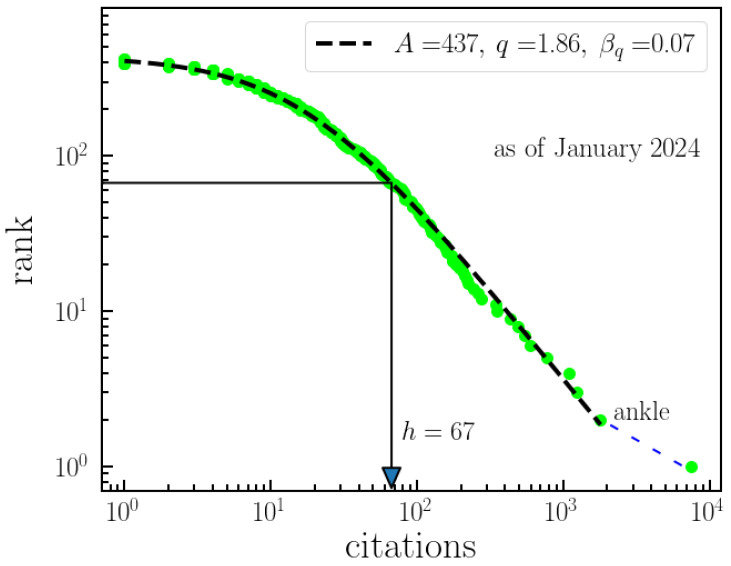
**Ranking of citations of Tsallis’ papers.** This figure presents an analysis of the citations received by papers authored by Tsallis. It illustrates the unnormalized decreasing cumulative distribution of citations for Tsallis’ papers, effectively fitted with a *q*-exponential distribution ($A\exp_{q}\left(-\beta_{q}x\right)$). The displayed index h=67 indicates the citation count at which the papers achieve an *h*-index of 67. This ranking offers insights into the impact and influence of Tsallis’ publications based on their citation counts. Furthermore, Tsallis’ seminal paper from 1988 stands out as an outlier, significantly contributing to the ankle point shown in the figure, which highlights its exceptionally high citation impact.

The citations of papers (co)authored by Constantino Tsallis are displayed in Figure 8, depicted as a Pareto-like plot, where the citations of each paper are plotted as a function of their respective rank, representing an unnormalized decreasing cumulative distribution. The log–log scale provides a clear visualization of the fitting of a *q*-exponential with parameters A=437, b=0.07, and q=1.86. A widely adopted measure is the *h*-index [26], proposed in 2005 to quantify the importance, significance, and impact of an individual researcher’s corpus. The definition of *h*-index is such that a researcher has an *h*-index of *n* if their top-*n* most-cited papers have been cited at least *n* times each, and the remaining papers have been cited fewer than *n* times each. Tsallis’ *h*-value, reaching a relatively large number of 67, is illustrated in Figure 8, indicating the average relevance of his scientific contributions. In contrast, the *q*-parameter reveals extraordinary contributions. Notably, the increase in citations of top-cited papers does not immediately affect the *h*-index, while the *q*-parameter increases. Tsallis’ top-ranked paper [15] appears as an outlier, akin to phenomena like highly energetic cosmic rays described as an “ankle” (see Figure 1 of [27]), where the ankle signifies extraordinarily highly cited papers.

## 4. Concluding Remarks

**Figure 9:**
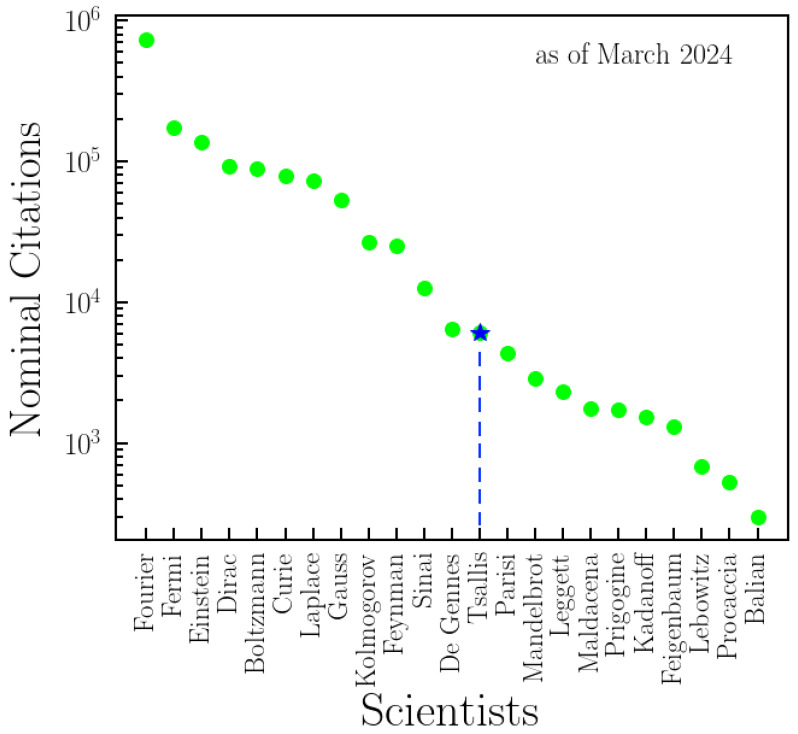
**Nominal citations in thermal physics: Comparison with titans.** A comparison of nominal citations is conducted for a non-exhaustive list of scientists with remarkable contributions to thermal physics throughout history and other relatively well-known scientists within the current statistical mechanics community. The numbers presented here are obtained from WoS by employing a search of each name in “All Databases”, including “Preprint Citation Index” in “Topic”. Tsallis is among the most-cited scientists in the field of thermal physics, providing insights into his relative impact and influence compared to other luminaries in the field.

Constantino Tsallis’ remarkable contributions to science and the *q*-Statistics are investigated by considering the meta-data of the associated articles and collaborators, which was collected from the Scopus database. We have presented scientometric indexes that express additional features to the difficult task of objectively, quantitatively, and unbiasedly classifying his activity. Furthermore, we have also compared the nominal citations of a non-exhaustive list of scientists with remarkable contributions to thermal physics throughout history and other relatively known scientists within the current statistical mechanics community (Figure 9). By nominal citation, we mean the appearance of the name of the scientist in the topic, title, or abstract, rather than in the authorship, of a cited paper, according to Web of Science. Scientists like K.E. Wilson, M.E. Fisher, and H.E. Stanley clearly belong to this group. However, their names are not included because of the very large number of homonyms. If the results we have found are replicated to other scientists and fields (especially for scientists with major contributions, for which fluctuations due to poor statistics tend to be minimized), we believe the characterization of scientific activity will be better described.

In conclusion, Constantino Tsallis has made significant contributions to the field of statistical physics, with 438 publications amassing a total of 28,000 citations, according to Scopus data (as of May 2024, 461 publications, according to Web of Science (All Databases) data; 44,146 citations according to Google Scholar citations data). He has also supervised and continues to supervise numerous students and colleagues, including the authors of this article. His love for science, a deep curiosity about nature, and friendly mentorship, coupled with wise advice, have consistently inspired us. This harmonious blend of motivation, passion, and enthusiasm for statistical physics continues to drive our collective efforts in the field.

## Data Availability

The data used in this study to analyze the contributions, collaborations, and citations of Professor C. Tsallis are sourced from Web of Science (WoS) and Scopus. The majority of the data were obtained from Scopus by searching “Constantino Tsallis” with Author ID 7006572244. Additional data were included to address gaps in the Scopus data, as detailed in the main text. Data for Figure 9, related to nominal citations in thermal physics, were downloaded from WoS using the keyword “Thermal Physics”. Researchers can access the original data through these databases. For detailed information or specific data requests, please contact the corresponding author.
